# TwinF interface inhibitor FP802 stops loss of motor neurons and mitigates disease progression in a mouse model of ALS

**DOI:** 10.1016/j.xcrm.2024.101413

**Published:** 2024-02-06

**Authors:** Jing Yan, Yu Meng Wang, Andrea Hellwig, Hilmar Bading

**Affiliations:** 1 Department of Neurobiology, Interdisciplinary Center for Neurosciences (IZN), Heidelberg University, 69120 Heidelberg, Germany

**Keywords:** glutamate neurotoxicity, excitotoxicity, extrasynaptic NMDA receptor, amyotrophic lateral sclerosis, neuroprotection, gene expression, biomarker, NMDAR/TRPM4 death signaling complex, TwinF interface inhibitor, FP802

## Abstract

Toxic signaling by extrasynaptic NMDA receptors (eNMDARs) is considered an important promoter of amyotrophic lateral sclerosis (ALS) disease progression. To exploit this therapeutically, we take advantage of TwinF interface (TI) inhibition, a pharmacological principle that, contrary to classical NMDAR pharmacology, allows selective elimination of eNMDAR-mediated toxicity via disruption of the NMDAR/TRPM4 death signaling complex while sparing the vital physiological functions of synaptic NMDARs. Post-disease onset treatment of the SOD1^G93A^ ALS mouse model with FP802, a modified TI inhibitor with a safe pharmacology profile, stops the progressive loss of motor neurons in the spinal cord, resulting in a reduction in the serum biomarker neurofilament light chain, improved motor performance, and an extension of life expectancy. FP802 also effectively blocks NMDA-induced death of neurons in ALS patient-derived forebrain organoids. These results establish eNMDAR toxicity as a key player in ALS pathogenesis. TI inhibitors may provide an effective treatment option for ALS patients.

## Highlights

• eNMDARs promote ALS disease progression via the NMDAR/TRPM4 death signaling complex
• TwinF interface inhibitor FP802 disrupts the NMDAR/TRPM4 death signaling complex
• FP802 is therapeutically effective in an ALS mouse model
• FP802 protects against NMDA-induced death in brain organoids from ALS patient iPSCs

## Graphical abstract

**Figure:**
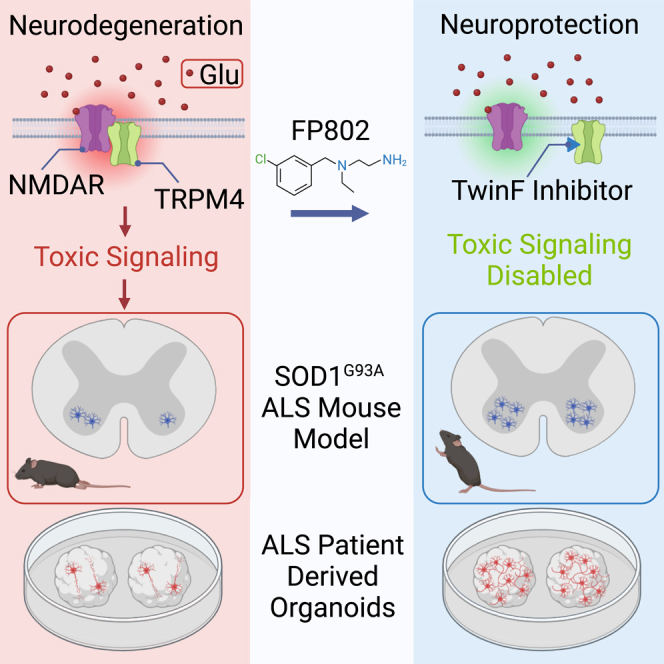

## Teaser

Yan et al. find that FP802, which provides neuroprotection by detoxifying eNMDARs through disruption of the NMDAR/TRPM4 complex, halts motor neuron loss in an ALS mouse model, reduces serum NfL levels, improves motor performance, and extends life expectancy. FP802 is also neuroprotective in brain organoids derived from ALS patients.

## Introduction

Amyotrophic lateral sclerosis (ALS) is the most frequent adult-onset human motor neuron disease. It affects both upper motor neurons in the cerebral cortex and lower motor neurons in the brain stem and spinal cord.^1^^,^^2^^,^^3^ ALS is untreatable, progresses rapidly, disables voluntary muscle movement, and causes death by respiratory failure. While in approximately 10% of affected individuals the disease is caused by mutations in one or several genes, for the majority of ALS cases, the origin of the disease is unknown.^1^^,^^2^^,^^3^^,^^4^^,^^5^ Both the inherited, familial forms of ALS and the much more common sporadic form share deficits in expression or function of excitatory amino acid transporters (EAATs). This leads to glutamate leakage from the synaptic cleft and increases extracellular glutamate concentrations to neurotoxic levels.^6^^,^^7^^,^^8^^,^^9^ NMDA receptors (NMDARs), in particular those located outside synaptic contacts that initiate toxic signaling, have been implicated as initiators of death signaling in ALS^5^^,^^10^^,^^11^ as well as in other neurodegenerative diseases.^12^^,^^13^ However, the precise role of extrasynaptic NMDARs (eNMDARs) in ALS remains largely unexplored. Despite the availability of potent NMDAR blockers,^14^ pharmacology studies face difficulties in their interpretation due to the dual function of NMDARs in the brain. eNMDARs are the primary mediator of glutamate-induced neuronal cell death,^11^^,^^12^^,^^13^^,^^15^^,^^16^ while synaptic NMDARs (sNMDARs) control synaptic plasticity-related events and cognitive functions.^17^ Classical NMDAR blockers do not distinguish between NMDARs located outside and inside the synapse and thus eliminate not only the toxic actions but also the physiological functions.^14^ This limits their value for animal studies of neurodegenerative diseases and their suitability as therapeutics in clinical trials.^18^^,^^19^ A recent investigation into the destructive nature of eNMDARs led to the discovery that their physical interaction with the transient receptor potential cation channel subfamily M member 4 (TRPM4) is responsible for their toxic signaling.^20^^,^^21^ This mechanistic insight provided the basis for the development of a class of small-molecule neuroprotective drugs, termed TwinF interface (TI) inhibitors. TI inhibitors disrupt the NMDAR/TRPM4 death signaling complex through binding to the TwinF domain, the TRPM4 interaction interface responsible for the NMDAR/TRPM4 complex formation.^20^^,^^21^ TI inhibitors selectively eliminate toxic signaling of eNMDARs while sparing the physiological functions of sNMDARs.^20^^,^^21^ In this study, we first explored the chemical space of compound 8, a prototype TI inhibitor. This yielded a safe and potent neuroprotectant, FP802, which was subsequently used to investigate the role of eNMDARs in the pathogenesis of ALS.

## Results

**Figure 1:**
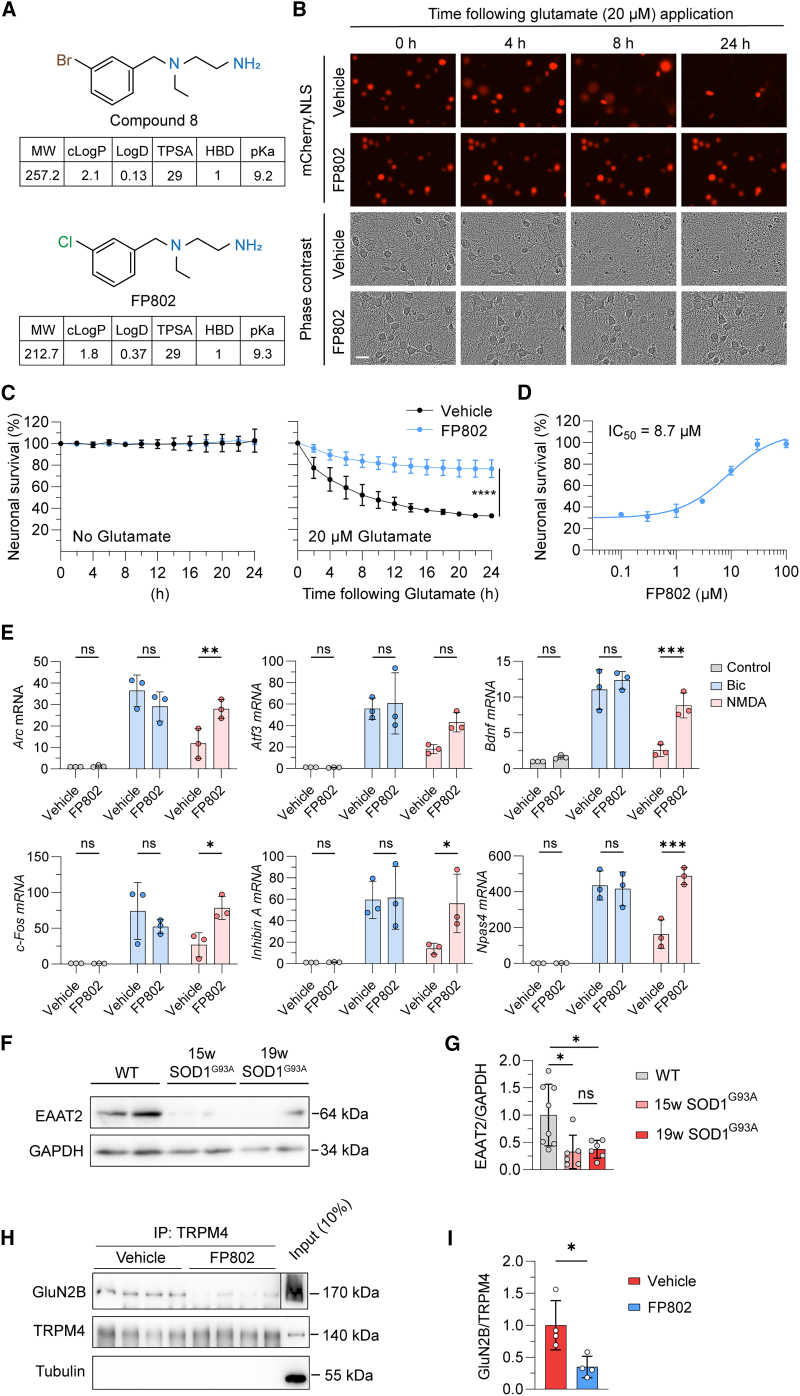
FP802 is neuroprotective, boosts gene expression, and disrupts the NMDAR/TRPM4 complex (A) Chemical structures and physicochemical parameters of compound 8 and FP802. MW, molecular weight; cLogP, calculated logarithm of partition coefficient; LogD, logarithm of distribution coefficient; TPSA, topological polar surface area; HBD, hydrogen bond donor; pKa, logarithm of acid dissociation constant. (B–D) FP802 protects primary mouse cortical neurons from glutamate neurotoxicity. (B) Representative time series images of primary cortical neurons, expressing mCherry-NLS, following glutamate insult. Scale bar, 20 μm. (C and D) Analysis of the percentage of nuclear mCherry-positive neurons with or without treatment with FP802 and with or without subsequent exposure to 20 μM glutamate, which is the EC_80_ of glutamate for death of cortical neurons. The IC_50_ of FP802 is 8.7 μM. Data represent means ± SD, n = 3; ∗∗∗∗p < 0.0001, unpaired t test. (E) FP802 restores impaired IEG expression induced by excitotoxic stimulation. Primary mouse cortical neurons were pre-incubated with FP802 (10 μM) or vehicle for 30 min before a 2-h treatment with either bicuculline (Bic, 50 μM) to induce action potential (AP) bursting or NMDA (20 μM) to induce excitotoxic insults. The mRNA levels of *Arc*, *Atf3*, *Bdnf*, *c-Fos*, *Inhibin beta A*, and *Npas4* were assessed by RT-qPCR. Data represent means ± SD, n = 3; ns: no significant difference, ∗p < 0.05, ∗∗p < 0.01, ∗∗∗p < 0.001, two-way ANOVA followed by Dunnett's multiple comparisons. (F and G) Immunoblot analysis of EAAT2 and GAPDH expression in the lumbar spinal cord of 19-week-old wild-type (WT) and SOD1^G93A^ mice. Data represent means ± SD, n = 6-8; ∗p < 0.05, Brown-Forsythe and Welch ANOVA test followed by Dunnett’s T3 multiple-comparisons test. (H and I) Co-immunoprecipitation with anti-TRPM4 antibodies of the NMDAR/TRPM4 complex from lumbar spinal cord lysates of animals treated with vehicle or FP802. In (H), compared to the GluN2B signals in the “Vehicle” and “FP802” lanes, the GluN2B signal in the “Input” lane originates from a shorter exposure time during digital imaging of the chemiluminescent signal. Data represent means ± SD, n = 4; ∗p < 0.05, unpaired t test.

TI inhibitors provide neuroprotection by disrupting a death signaling complex consisting of eNMDARs and TRPM4 that is primarily responsible for NMDAR-mediated glutamate neurotoxicity.^20^ Compound 8, the prototype TI inhibitor, virtually eliminates the pathological triad characteristic of toxic signaling of eNMDARs,^20^ which includes loss of structural integrity of neurons, CREB shut-off and transcriptional deregulation, and mitochondrial dysfunction.^11^ Compound 8 is a potent neuroprotectant both in neuronal cell culture and *in vivo* in mouse models of stroke and retinal degeneration.^20^ Because compound 8 contains a phenyl bromide,^20^ it may upon breakdown liberate bromide or methyl bromide, which could cause irritations and poisoning.^22^^,^^23^ This raises concerns about long-term *in vivo* applicability of compound 8 and prompted us to generate variants of compound 8 lacking bromide. This yielded FP802, a phenyl chloride containing derivative of compound 8 with similar physicochemical parameters (Figure 1A) and potent protective activity against glutamate-mediated neurotoxicity (IC_50_ 8.7 μM; Figures 1B–1D). Similar to compound 8, FP802 eliminated the transcriptional shut-off induced by eNMDARs and boosted the NMDA bath application-induced expression of the immediate-early genes (IEGs) *Atf3*, *Arc*, *Bdnf*, *cFos*, *Inhibin beta A*, and *Npas4* to reach levels that were comparable to those achieved by bicuculline-induced action potential bursting (Figure 1E), which activates transcription-promoting signaling by sNMDARs.^10^^,^^16^^,^^20^ Electrophysiology experiments revealed that similar to compound 8,^20^ FP802 did not block NMDARs expressed heterologously in HEK293 cells (IC_50_ > 250 μM for both, GluN1/GluN2A and GluN1/GluN2B) (Figure S1). In addition, we investigated pharmacology safety of FP802 using radioligand binding assays for 30 common targets and found no significant interaction with any target tested (Table S1).

**Figure 2:**
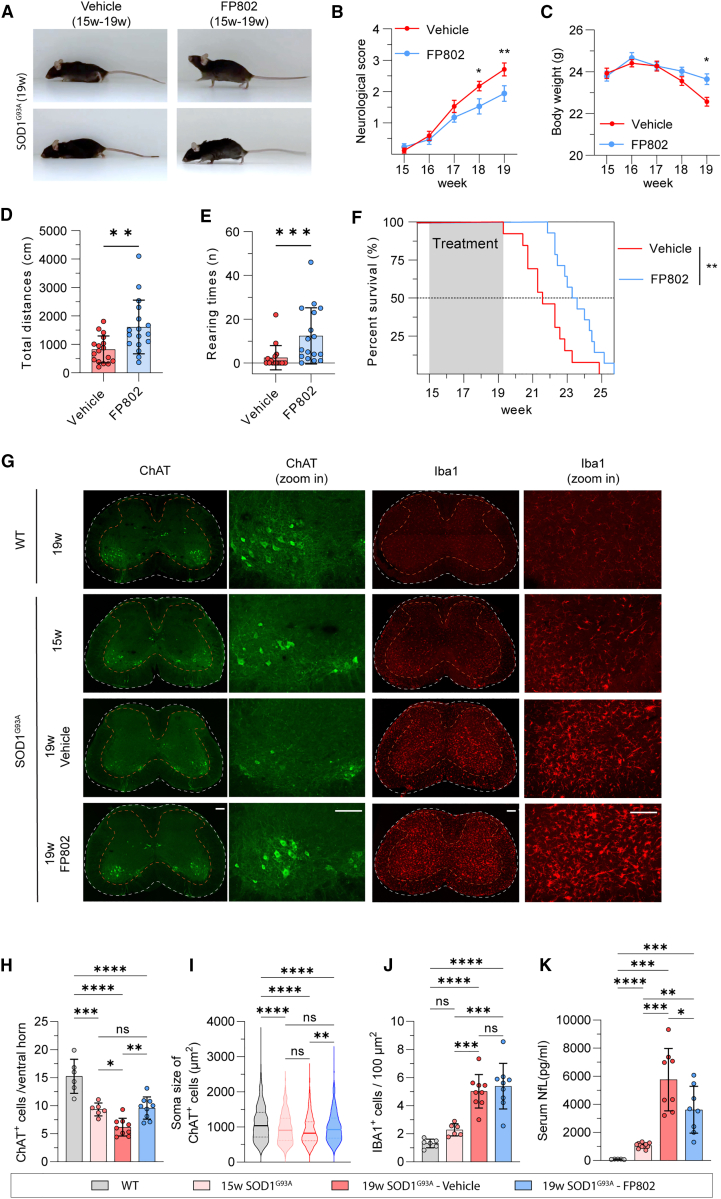
FP802 improves motor performances, extends lifespan, and prevents motor neuron loss in SOD1^G93A^ mice (A) Representative images of SOD1^G93A^ mice at 19 weeks following 4 weeks of treatment with vehicle or FP802. (B–E) Neurological scores, body weight changes, total travel distances, and rearing times of SOD1^G93A^ mice following vehicle or FP802 treatment (n = 17 mice in each group). (B and C) Data represent means ± SEM; ∗p < 0.05, ∗∗p < 0.01, ∗∗∗p < 0.001, ∗∗∗∗p < 0.0001, two-way RM ANOVA followed by Šidák’s multiple comparisons. (D and E) Data represent means ± SD; ∗∗p < 0.01, ∗∗∗p < 0.001, Mann-Whitney test. (F) The lifespan of SOD1^G93A^ mice with or without treatment with FP802 for 4 weeks starting at week 15;n = 13–14 mice in each group, ∗∗p < 0.01, Gehan-Breslow-Wilcoxon test. (G) Representative images of L3–L5 lumbar spinal cord slices of 19-week-old wild type (WT), 15-week-old SOD1^G93A^ mice, and of 19-week-old SOD1^G93A^ mice with vehicle or FP802 treatment starting at week 15, stained using antibodies to ChAT and Iba1. Scale bar, 200 μm. (H) Quantification of ChAT-positive cells in the ventral horn of lumbar spinal cord (L3–L5). Data represent means ± SD, n = 6–9 mice in each group; ns: no significance, ∗p < 0.05, ∗∗p < 0.01, ∗∗∗p < 0.001, ∗∗∗∗p < 0.0001, one-way ANOVA followed by Tukey’s multiple-comparisons test. (I) Distribution of the soma size of ChAT-positive cells from (H) shown in violin plot. Data represent means ± SD, n = 500–900 ChAT-positive cells from 6–9 mice in each group; ns: no significance, ∗∗p < 0.01, ∗∗∗∗p < 0.0001, one-way ANOVA followed by Tukey’s multiple-comparisons test. (J) Quantification of signal intensity of Iba1-positive cells in the ventral horn of lumbar spinal cord (L3–L5). Data represent means ± SD, n = 6–9 mice in each group; ns: no significance, ∗∗∗p < 0.001, ∗∗∗∗p < 0.0001, one-way ANOVA followed by Tukey’s multiple-comparisons test. (K) Quantification of the neurofilament light chain (NfL) levels in the serum. Data represent means ± SD, n = 5–11 mice in each group; ∗p < 0.05, ∗∗p < 0.01, ∗∗∗p < 0.001, Brown-Forsythe and Welch ANOVA followed by unpaired t test with Welch’s correction multiple comparison.

We next used FP802 in the transgenic SOD1^G93A^ mouse model of ALS that expresses mutant human SOD1 with a glycine to alanine substitution at position 93 and whose general features resemble ALS in humans.^24^^,^^25^ SOD1^G93A^ mice represent a widely accepted animal model used extensively in ALS drug discovery to evaluate the efficacy of compounds now in clinical trials, which include masitinib, pridopidine, fasudil, guanabenz, and AMX0035.^26^^,^^27^^,^^28^^,^^29^^,^^30^^,^^31^^,^^32^ We first confirmed using immunoblot analysis the reduction of EAAT2/GLT-1 expression in the spinal cord of SOD1^G93A^ mice (Figures 1F and 1G). Deregulation of glutamate uptake systems may lead to an increase in toxic eNMDAR signaling, which could be the cause of motor neuron death. To test this hypothesis, we applied FP802 subcutaneously to SOD1^G93A^ mice using an implanted ALZET osmotic mini pump to achieve a steady release of the drug. FP802 readily crossed the mouse blood-spinal cord barrier and reached concentrations in the spinal cord of 7.54 ± 4.79 nmol/g (n = 3), corresponding to approximately 3–12 μM FP802, when applied at 40 mg/kg/day for 2 weeks. Co-immunoprecipitation experiments using lysates from mouse spinal cord revealed that in samples from FP802-treated mice, the interaction of TRPM4 with the NMDAR subunit GluN2B was disrupted (Figures 1H and 1I). Following successful deactivation of the NMDAR/TRPM4 death signaling complex by FP802 *in vivo*, we next assessed potential therapeutic benefits of FP802 in SOD1^G93A^ mice. To implement an experimental design that resembles a clinical situation, we started treatment of SOD1^G93A^ mice with FP802 at the time of onset of motor deficits, which was around week 15.^33^^,^^34^ SOD1^G93A^ mice treated with FP802 for the subsequent 4 weeks had significantly better neurological scores and lower body weight losses compared to control-vehicle-treated animals (Figures 2A–2C). Moreover, SOD1^G93A^ mice had a significantly improved motor performance based on the total distances traveled in an open field and showed frequent rearing behavior during which the animals raise their forelimbs from the ground putting their entire weight on their hind legs (Figures 2D and 2E; Videos S1 and S2).

Video S1. Representative video of two 19-week-old SOD1G93A mice in the vehicle group, related to Figure 2

Video S2. Representative video of two 19-week-old SOD1G93A mice in the FP802 group, related to Figure 2

FP802 treatment also significantly extended the lifespan of SOD1^G93A^ mice (Figure 2F; survival median increased from 151 to 164 days), which may be due to the enhanced survival of spinal motor neurons. To investigate this, we assessed the death of spinal motor neurons in FP802-treated SOD1^G93A^ mice. Immunohistochemical analyses revealed that already at disease onset at 15 weeks, i.e., before the start of FP802 administration, the number of lumbar spinal motor neurons identified by the choline acetyltransferase (ChAT) marker was significantly lower in SOD1^G93A^ mice compared to healthy wild-type (WT) littermates (Figures 2G and 2H). We also observed at 15 weeks of age a shift in the size distribution of lumbar spinal motor neurons toward smaller soma sizes in SOD1^G93A^ mice compared to WT (Figure 2I). In control-vehicle-treated SOD1^G93A^ mice, the number of lumbar spinal motor neurons further decreased dramatically within the subsequent 4 weeks, whereas in FP802-treated animals, no additional death was observed at week 19 (Figures 2G and 2H). In addition, size distribution analysis revealed that at week 19, the soma sizes of lumbar spinal motor neurons from FP802-treated SOD1^G93A^ mice were larger than the soma sizes of lumbar spinal motor neurons from control-treated SOD1^G93A^ mice (Figure 2I). The neuroinflammatory response associated with the spinal cord pathology in SOD1^G93A^ mice, assessed using the microglia marker Iba1, was not affected by FP802 treatment (Figures 2G and 2J). However, neuroprotection of spinal motor neurons by FP802 was accompanied by a reduction of serum levels of neurofilament light (NfL) chain, a biomarker used in ALS patients to monitor disease progression (Figure 2K).^35^^,^^36^ As FP802 had no significant effect on EAAT/GLT-1 expression (Figure S2), these results strongly suggest that toxic eNMDAR signaling is an important driver of motor neuron degeneration and death in SOD1^G93A^ ALS mice. To assess possible adverse effects of FP802 treatment on liver, kidney, and heart, we monitored a panel of serum parameters and blood cell counts. The values measured did not differ significantly between untreated and FP802-treated animals (Tables S2 and S3).

**Figure 3:**
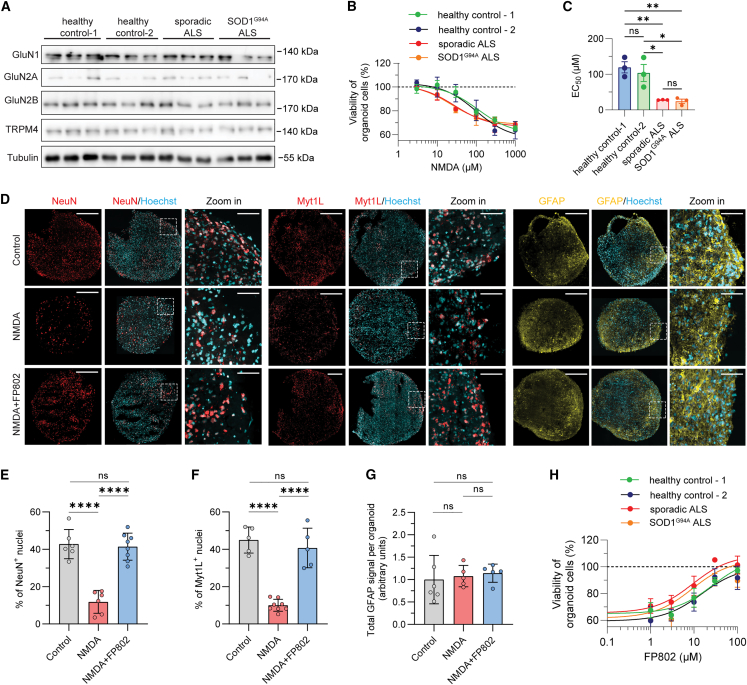
FP802 protects human brain organoids generated using iPSCs from ALS patients or healthy controls against NMDA-induced neurotoxicity (A) Immunoblot analysis of GluN1, GluN2A, GluN2B, TRPM4, and tubulin expression in human iPSC-derived forebrain organoids. For each iPSC-line-derived organoid, 3 independently generated lysates from 2 to 3 organoids each were analyzed. (B) Nonlinear fit of viability of forebrain organoid cells following NMDA treatment. The EC_50_ value was calculated with the agonist concentration plotted against response (three parameters). Data represent means ± SEM, n = 3 with 2–3 organoids in each experiment. (C) Statistical analysis of EC_50_ values from (B). Data represent means ± SD; ns: no significance, ∗p < 0.05, ∗∗p < 0.01, one-way ANOVA followed by Tukey’s multiple-comparisons test. (D–G) FP802 protects post-mitotic neurons in forebrain organoids generated from sporadic ALS patient-derived iPSCs against NMDA-induced neurotoxicity. (D) Representative images of untreated organoids (Control) and organoids treated with NMDA (300 μM for 24 h) without or with pre-treatment for 30 min with 30 μM FP802 and stained with Hoechst 33258 and using antibodies to NeuN, Myt1L, and GFAP. Scale bar, 500 μm. Scale bar in zoomed-in images, 50 μm. (E–G) Quantification of the immunostainings in (D). NeuN-positive (NeuN^+^) cells (E) and Myt1L-positive (Myt1L^+^) cells (F) were counted in confocal section z stacks of whole organoids (n = 5–8 organoids from 2 batches) and expressed as a percentage of total Hoechst-stained nuclei. GFAP staining (G) was quantified by measuring the fluorescent signals of z stacks of confocal sections of whole organoids (n = 5–6 organoids from 2 batches) and expressed as arbitrary units. Data represent means ± SD; ns: no significance, ∗∗∗∗p < 0.0001, one-way ANOVA followed by Tukey’s multiple-comparisons test. (H) Nonlinear fit of protection by FP802 of forebrain organoids against NMDA-induced neurotoxicity; NMDA, 300 μM. IC_50_ values (in μM: healthy control-1: 26; healthy control-2: 15; sporadic ALS: 9.3; SOD1^G94A^ ALS: 9.0) were calculated with FP802 concentration versus response (three parameters). Data represent means ± SEM, n = 3 with 2–3 organoids in each experiment.

We finally investigated the translatability of the animal data to human neurons and used induced pluripotent stem cells (iPSCs) derived from patients with either a sporadic or the SOD1^G94A^ form of ALS that we differentiated into human forebrain organoids (G94A refers to the same mutation as G93A but with different nomenclature; see https://www.jax.org/strain/004435 for details). Organoids generated from all iPSC lines used in this study express the NMDAR subunits, GluN1, GluN2A, GluN2B, and TRPM4 (Figure 3A). We found that organoids derived from ALS patients with either the sporadic or the SOD1^G94A^ form of the disease were more sensitive to NMDAR toxicity than human organoids generated using iPSCs from healthy controls (Figures 3B and 3C). To identify the cell population vulnerable to NMDAR toxicity, ALS patient-derived organoids were immunostained with antibodies to the nuclear proteins, NeuN and myelin transcription factor 1-like (Myt1L) protein, which identify post-mitotic neurons, and with antibodies to the astrocyte marker, glial fibrillary acidic protein (GFAP). Similar to the results obtained in a previous study in which organoids from healthy donors were analyzed,^37^ we found that the number of NeuN-positive and Myt1L-positive post-mitotic neurons was dramatically reduced in ALS patient-derived organoids after NMDA exposure, while GFAP expression remained unchanged (Figures 3D–3G). FP802 effectively blocked, in a dose-dependent manner, the NMDA-induced death of neurons in ALS patient-derived organoids (Figures 3D–3H). These results indicate that toxic eNMDAR signaling is an evolutionary conserved neuronal death process that can be effectively inhibited by the TI inhibitor FP802 in human neurons from ALS patients.

## Discussion

The results of this study have far-reaching clinical implications. While the SOD1^G93A^ mouse model has its limitations and is not necessarily a predictor of results obtained with other animal models of ALS or with ALS patients, our study takes a comprehensive approach by incorporating iPSC-derived neurons from ALS patients with the SOD1^G94A^ mutation or the sporadic form of the disease. The consistent neuroprotective effects of FP802 across these diverse human neuronal populations provide solid evidence that our findings have relevance beyond the SOD1^G93A^ mouse model of ALS. Blocking the TI with FP802 therefore has potential to be developed into an effective therapy for ALS. Differently from NMDAR blockers, FP802 inhibits ongoing eNMDAR-mediated glutamate neurotoxicity in ALS while leaving unaltered the vital physiological functions of sNMDARs.^20^^,^^21^ This distinction makes FP802 a promising treatment option for both familial and sporadic forms of ALS that share glutamate neurotoxicity as pathomechanism.^4^^,^^6^^,^^7^^,^^38^ Thus, TI inhibitors may exhibit broad applicability and, as such, offer a compelling alternative to the recently developed antisense oligonucleotide therapy,^39^ which is tailored exclusively for a small subset of ALS patients with hereditary, monogenic forms of the disease caused, for example, by gain-of-function mutations in SOD1 or C9orf72.^40^^,^^41^^,^^42^ TI inhibitors may substantially mitigate suffering of ALS patients and extend their life expectancy.

### Limitations of the study

The study is constrained by the challenge of accurately modeling ALS in human organoids. Despite ongoing advancements in the protocols for generating human brain organoids, replication of the precise synaptic circuits associated with ALS remains challenging. This limitation is compounded by the difficulty in generating human motor neurons within organoids that express fully functional NMDARs crucial for studying glutamate neurotoxicity associated with ALS. In our study, we opted for an alternative approach, utilizing human forebrain organoids generated with iPSCs derived from both ALS patients and healthy donors. The implemented cell culture protocol yielded human forebrain organoids that exhibited sensitivity to NMDA-induced neurotoxicity, enabling us to assess the effectiveness of FP802 in human neurons. A crucial next step toward clinical translation is to demonstrate the neuroprotective effects of FP802 in mature human motor neurons with fully functional NMDARs within an ALS organoid system.

## STAR★Methods

### Key resources table

**Table:** 

REAGENT or RESOURCE	SOURCE	IDENTIFIER
**Antibodies**		

Mouse monoclonal anti-EAAT2	Proteintech	Cat#ABN90; RRID: AB_2882391
Goat polyclonal anti-ChAT	Millipore	Cat#AB144P; RRID: AB_2079151
Goat polyclonal anti-Iba1	Abcam	Cat#Ab5076; RRID: AB_2224402
Rabbit monoclonal anti-GAPDH	Cell Signaling Technology	Cat#2118; RRID: AB_561053
Rabbit polyclonal anti-TRPM4	Millipore	Cat#ABN418; RRID: AB_2891307
Rabbit polyclonal anti-TRPM4	Yan et al.^20^	N/A
Rabbit polyclonal anti-GluN1	Abcam	Cat#Ab17345; RRID: AB_776808
Rabbit polyclonal anti-GluN2A	Millipore	Cat#AB1555; RRID: AB_2112325
Rabbit polyclonal anti-GluN2B	Novus	Cat#NB300-106; RRID: AB_10000537
Rabbit polyclonal anti-Myt1L	Dr. Moritz Mall, DKFZ, Heidelberg	N/A
Mouse monoclonal anti-NeuN	Biolegend	Cat#834501; RRID: AB_2564991
Mouse monoclonal anti-GFAP	Cell Signaling Technology	Cat#3670S; RRID: AB_561049

**Bacterial and virus strains**		

pAdDeltaF6	Zhang et al.^43^	Addgene#112867
pRV1	Zhang et al.^43^	N/A
pH21	Zhang et al.^43^	N/A
pAAV-hSyn-mCherry.NLS	This paper	N/A

**Chemicals, peptides, and recombinant proteins**		

Neurobasal™-A Medium	Gibco™	Cat#10888022
B-27™ Supplement (50x), serum-free	Gibco™	Cat#17504044
GlutaMAX™ Supplement	Gibco™	Cat#35050038
Penicillin-Streptomycin	Gibco™	Cat#15070063
Opti-MEM	Gibco™	Cat#31985047
Rat serum	Biowest	Cat#S2150
Glutamate	Tocris	Cat#0218
Na_2_SO_4_	Sigma Aldrich	Cat#S5640
K_2_SO_4_	Sigma Aldrich	Cat#P8541
CaCl_2_	Sigma Aldrich	Cat#C7902
MgCl_2_	Sigma Aldrich	Cat#M2393
Kynurenic acid	Sigma Aldrich	Cat#K3375
Papain latex	Cell Systems	Cat#LS003127
Poly-D-lysine hydrobromide	Sigma Aldrich	Cat#P7886
Laminin	Sigma Aldrich	Cat#L2020
Trypsin inhibitor	Sigma Aldrich	Cat#T9253
NMDA	Tocris	Cat#0114
Tris base	Merck	Cat#T1503
NaCl	Merck	Cat# S167
EDTA	AppliChem	Cat#A3553
Triton X-100	Merck	Cat#108603 CAS:9036-19-5
Glycerol	VWR	Cat#24387.292 CAS:56-81-5
Pierce™ BCA Protein Assay Kits	Thermo Scientific™	Cat#23225
cOmplete™, EDTA-free Protease Inhibitor Cocktail	Roche	Cat#11873580001
Pierce™ Protein A/G Magnetic Beads	Thermo Scientific™	Cat#88803
Paraformaldehyde	Merck	Cat#158127
Corning Matrigel Matrix	Corning	Cat#356255
DMEM/F12	Thermo Scientific™	Cat#31330038
mTeSR™ Plus	STEMCELL Technologies	Cat#100-0276
Normocin	Invivogen	Cat#ant-nr-1
N-2 Supplement	Thermo Scientific™	Cat#17502048
Dispase	STEMCELL Technologies	Cat#07923
Accutase	STEMCELL Technologies	Cat#07920
B27 without Vitamin A	Thermo Scientific™	Cat#12587010
Dorsomorphin	Tocris	Cat#3093 CAS:1219168-18-9
Recombinant Noggin	R&D Systems	Cat#719-NG
SB431542	Tocris	Cat#1614 CAS:301836-41-9
β-Mercaptoethanol	Thermo Scientific™	Cat#21985023 CAS:60-20-4
IWP-2	Sigma Aldrich	Cat#681671 CAS:686770-61-6
Cyclopamine	Tocris	Cat#3093 CAS:1219168-18-9
Recombinant human EGF	Thermo Scientific™	Cat#PHG0311L
Recombinant human FGF-basic	Peprotech	Cat#100-18B
Recombinant human BDNF	Peprotech	Cat#450-02
Recombinant human NT-3	Peprotech	Cat#450-03
BrainPhys™ Neuronal Medium	STEMCELL Technologies	Cat#05790
FP802	Wuxi AppTec/Chemspace	CAS:61694-81-3
Propylene Glycol	Sigma Aldrich	Cat#P4347 CAS:57-55-6
Hoechst 33258	Sigma Aldrich	Cat#14530 CAS:23491-45-4
Mowiol 4-88	Sigma Aldrich	Cat#475904 CAS:9002-89-5

**Critical commercial assays**		

Automated Patch Clamp	Charles River Laboratories, UK	Custom-made, Yan et al.^20^
Pharmacology safety screening of FP802	Eurofins Panlabs Discovery Services Taiwan, Ltd	Assay ID in Table S1
Hematology and Clinical Chemistry	IDEXX Laboratories, DE	Assay ID in Table S2/3
Neurofilament light chain measurement with Simoa® NF-light™ Advantage Kit (Quanterix Cat #103186)	PBL Assay Science, US	N/A

**Experimental models: Cell lines**		

HEK293 cells	Stratagene	Cat#240073 RRID:DVDL_6871
Healthy control – 1, iPSC	Bauersachs et al.^37^	D1, Dr. Utikal, DKFZ, Heidelberg
Healthy control – 2, iPSC	Bauersachs et al.^37^	HD6, Dr. Utikal, DKFZ, Heidelberg
Sporadic ALS, iPSC	Cedars-Sinai	CS2XWCiALS-nxx
SOD1G94A ALS, iPSC	Cedars-Sinai	CS2RJViALS-nxx

**Experimental models: Organisms/strains**		

C57/Bl6, for primary cortical cultures	CRL	N/A
SOD1^G93A^ transgenic mice	Jackson Laboratory	Line#004435

**Oligonucleotides**		

Atf3	Taqman	Mm00476032_m1
Arc	Taqman	Mm00479619_g1
Bdnf	Taqman	Mm00432069_m1
cFos	Taqman	Mm00487425_m1
Inhibin beta A	Taqman	Mm00434338_m1
Npas4	Taqman	Mm00463644_m1

**Software and algorithms**		

Sygnis tracking software for Open Field analysis	INBC, DE	N/A
GraphPad Prism	N/A	RRID:SCR_002798
SAS	N/A	RRID: SCR_008567
ImageJ	N/A	RRID:SCR_003070

### Resource availability

#### Lead contact

Further information and requests for resources and reagents should be directed to and will be fulfilled by the lead contact, Hilmar Bading (Bading@nbio.uni-heidelberg.de).

#### Materials availability

This study did not generate new unique reagents except for FP802.

FP802 generated in this study will be made available on request, but we may require a payment and/or a completed Materials Transfer Agreement if there is potential for commercial application.

#### Data and code availability

(1) All data reported in this paper will be shared by the lead contact upon request.
(2) This paper does not report original code.
(3) Any additional information required to reanalyze the data reported in this paper is available from the lead contact upon request.

### Experimental model and study participant details

#### Animals

Heterozygous SOD1^G93A^ transgenic mice expressing human SOD1^G93A^ mutant on a C57BL/6 background (Jackson Laboratory, 004435, RRID:IMSR_JAX:004435) were used in this study.^24^^,^^34^ Heterozygosity was maintained by mating heterozygous transgenic males with C57BL/6 wild-type females. The animals were provided by the interdisciplinary neurobehavioral core (INBC) at Heidelberg University. Only male wild-type mice and male transgenic mice were used in the experiments. They were housed in groups (maximally three mice/cage) in the INBC, where all behavior experiments were performed. All mice were kept in standard cages (15 cm × 21 cm × 13.5 cm) on a 12:12 h light:dark cycle with *ad libitum* access to food, water, and nesting material. The treatments were randomly allocated to mice within litter blocks. The experimenter was blinded to the experimental group from the implantation until all analyses were done.

A total of 83 heterozygous SOD1^G93A^ and 22 wild-type littermates were employed in this study. Eight WT and 12 SOD1^G93A^ mice were used to determine the EAAT2 protein levels in the spinal cord. Three SOD1^G93A^ mice were treated with FP802 to explore the concentration of FP802 in the spinal cord. Four SOD1^G93A^ mice were used to test the tolerability of mice to subcutaneously ALZET osmotic pump (type 2004) implantation with FP802 that allowed a continuous and constant supply of compound to the animals for 4 weeks. Eight wild-type mice were used to explore the disruption of the NMDAR/TRPM4 death signaling complex by FP802. In total, 52 SOD1^G93A^ mice were treated with FP802 or vehicle via ALZET osmotic pump from week 15 for 30 days (four cohorts). It was not possible to extend the treatment beyond the 30 days application period because the poor health state of many animals was incompatible with the anesthesia required for surgical implantation of a second osmotic pump. The number of animals used for experiments was specified in the figure legends. One animal was excluded from the lifespan analysis because it showed an abnormally delayed disease onset and prolonged survival.^33^ In addition, six wild-type littermates and 12 SOD1^G93A^ mice were used at week 19 and week 15, respectively, for the analysis of motor neuron survival and serum NfL levels. For the analysis of motor neuron survival, three mice (two from wild-type littermates, one from vehicle-treated SOD1^G93A^ mice) were excluded from the motor neuron survival because of the loss of sample during preparation.

All animal procedures were carried out according to the German guidelines for the care and use of laboratory animals and in accord with the European Community Council Directive 2010/63/EU. This study was approved by the animal care and use committee, Regierungspräsidium Karlsruhe, Referat 35, Germany, AZ 35.9185.81/G-190/19. The authors declare that all experiments conformed to all relevant local regulatory standards.

#### Primary neuronal cultures

Primary mouse cortical neurons were prepared as previously described.^43^^,^^44^ Briefly, brain cortices from neonatal mice (C57/Bl6, RRID:MGI:7264769) were removed and cut into small pieces in the dissociation medium (in mM: 81.8 Na_2_SO_4_, 30 K_2_SO_4_, 15.85 MgCl_2_, 0.25 CaCl_2_, 1.0 HEPES, 1050.0 kynurenic acid; 0.2% phenol red and 0.36% glucose). The tissues were then digested with papain latex (10 units/mL) in the dissociation medium supplemented with 0.45 mg/mL L-cysteine for two times of 15 min in a 37°C water bath with stirring at 15 rpm. Then the tissue was washed twice with the dissociation medium and incubated with trypsin inhibitor (0.01 g/mL) in dissociation medium for 3 × 5 min. The samples were then triturated into single cell solution and plated at a density of 0.25 × 10^6^ cell per well in 24-well plates, which have been pretreated with poly-D-lysine (26.7 mg/μL) and laminin (6.7 mg/μL). Neurons were maintained in neurobasal-A medium supplemented with 1% rat serum, 1× B27 supplement, 1× L-GlutaMAX, and 1× penicillin/streptomycin at 37°C, 5% CO_2_ and 95% humidity. On day *in vitro* (DIV) 3, 2.8 μM cytosine beta-D-arabinofuranoside hydrochloride was added to the cultures to stop proliferation of non-neuronal cells. Primary neurons plated in 24 well plates were infected with 0.25 × 10^9^ rAAV-hSyn-mCherry-NLS viral particles on DIV3. Half medium change was done with medium without rat serum every two to three days starting on DIV8.

#### HEK293 cells

HEK293 cells were cultured with high glucose DMEM supplemented with 10% FBS, 1× penicillin and streptomycin, 1× non-essential amino acids, and 1× sodium pyruvate at 37°C, 5% CO_2_, and 95% humidity.

#### Human iPSC-derived forebrain organoids

The generation of human-iPSC-derived forebrain organoids was carried out in accordance with the regulations of Heidelberg University. The sporadic ALS iPSC line and the SOD1^G94A^ ALS iPSC line were purchased from Cedars-Sinai (CS2XWCiALS-nxx and CS2RJViALS-nxx). The healthy control iPSC lines were from Dr. Jochen Utikal (DKFZ, Germany). Forebrain organoids were generated and maintained as described previously.^37^^,^^45^ Briefly, hiPSCs were dissociated into single cells by Accutase and aggregated in round bottom 96-microwell plates with 5 min centrifugation at 600 rpm. On the second day, hiPSC aggregates were collected and transferred into low attachment 35 mm dishes (24 aggregates/dish) and kept in neural induction medium (DMEM/F12 medium supplemented with 1× penicillin/streptomycin, 1× Normocin, 1× GlutaMAX, 1× N-2 supplement, 2× B27 without vitamin A, 500 nM dorsomorphin, 50 ng/mL recombinant human noggin, 10 μM SB431542, 2 μM IWP-2, 1 μM cyclopamine, and 0.1% β-mercaptoethanol) for 3 days with a daily medium change. Recombinant human FGF (10 ng/mL) was present in neural induction medium from day 4 to day 7 with one medium change on day 5.5. On day 7, neural induction medium was changed to proliferation medium (neurobasal medium supplemented with 1× GlutaMAX, 1× penicillin/streptomycin, 1× Normocin, 1× B27 without vitamin A, 10 ng/mL recombinant human FGF, 10 ng/mL recombinant human EGF, 2 μM IWP-2, and 1 μM cyclopamine) for two weeks with medium change every 2–3 days. In week 4–5, the proliferation medium was changed to differentiation medium (neurobasal medium supplemented with 1× GlutaMAX, 1× penicillin/streptomycin, 1× Normocin, 10 ng/mL recombinant human BDNF, and 10 ng/mL recombinant human NT-3) with medium change every 2–3 days. From week 6, the organoids were kept in the maturation medium (BrainPhys medium supplemented with 1× GlutaMAX, 1× penicillin/streptomycin, 1× Normocin) until week 18–20 when they were used for experiments.

### Method details

#### Synthesis of FP802

FP802 was synthesized in three steps. Chemicals are given as smiles. First, ClC1=CC(C=O)=CC=C1 is added to NCCNC(OC(C)(C)C)=O in presence of sodium cyanoborohydride and methanol to generate ClC1=CC(CNCCNC(OC(C)(C)C)= O)=CC=C1. Second, the ethyl group is added with 10 eq acetaldehyde in the presence of sodium cyanoborohydride and methanol at 25°C to generate ClC1=CC(CN(CC)CCNC(OC(C)(C)C)=O)=CC=C1. Third, the protective tert-butyloxycarbonyl (Boc) is removed by HCl and ethyl acetate to generate FP802 (ClC1=CC(CN(CC)CCN)=CC=C1) in a salt format with 2 HCl. Mass spectrometry revealed purity of FP802 > 99.5%.

#### Glutamate neurotoxicity assay in primary cortical neurons

On DIV10, vehicle (DMSO) or FP802 were added to the primary cortical cultures 30 min prior to glutamate insult. Images were acquired following the glutamate application for 24 h with 2 h intervals, where 9 images were obtained via a 20x objective for each condition at each time point with IncuCyte S3 Live-Cell Analysis System (Sartorius AG, Germany). Survival of neuronal cells (%) was quantified by analyzing the number of mCherry-positive nuclei with the Basic Analyzer of the IncuCyte 2021 software using size (50–150 μm^2^) and average intensity (>10) as selection criteria.

#### Recombinant adeno-associated virus (rAAV) and expression vectors

2-3 h before the transfection, the culture medium was changed to IMEM supplemented with 5% FBS. Then the HEK293 cells were transfected using pAdDeltaF6, pRV1, pH21, and pAAV-hSyn-mCherry-NLS (molar ratios: 2:0.75:0.75:1); for each 15 cm dish, 25 μg plasmids in total were used and a calcium phosphate-based transfection medium (25 mM HEPES, 140 mM NaCl, 1.5 mM Na_2_HPO_4_, and 165 mM CaCl_2_). 16 h after transfection, the medium was changed to normal culture medium and cells were washed and harvested in phosphate-buffered saline (PBS). The rAAV containing HEK293 cell pellets were resuspended with 150 mM NaCl/10 mM Tris (pH 8.5) with 0.5% sodium deoxycholate and 50 units/ml Benzonase at 37°C for 1 h. The rAAV particles were purified via heparin columns. Briefly, the heparin column was pre-washed with 10 mL 150 mM NaCl/10 mM Tris before loading with 50 mL HEK293 cell lysate containing rAAV particles, then the column was washed with 1 mL of 200 mM NaCl/10 mM Tris and 300 mM NaCl/10 mM Tris to get rid of unspecific binding. The rAAV particles were collected in 1.5 mL 400 mM NaCl/10 mM Tris, 3.0 mL 450 mM NaCl/10 mM Tris, and 1.5 mL 500 mM NaCl/10 mM Tris, sequentially. Finally, the rAAV particles were concentrated with Amicon Ultra-4 centrifugal filter devices 100K NMWL, sterile filtered (0.22 μm), and stored in PBS at 4°C. The titer of rAAV particles (genome copies/ml) was determined with quantitative real-time PCR with primers designed to woodchuck hepatitis virus posttranslational regulatory element (WPRE) as described previously^43^

#### Immunoprecipitation and immunoblot analysis

All procedures for immunoprecipitation were carried out at 4°C as described before.^20^ Lumbar spinal cords were dissected from mice two weeks after ALZET osmotic pump (1002) implantation. Tissues were homogenized and lysed in immunoprecipitation buffer (10 mM Tris, pH 8.0, 150 mM NaCl, 1 mM EDTA, 1% Triton X-100, 10% glycerol) containing EDTA-free protease inhibitor cocktail (Roche) for 60 min. The lysate was centrifuged for 10 min at 1200 × g to remove cell debris and nuclei and then pre-cleared with anti-rabbit-IgG and protein A/G magnetic beads (Pierce). Protein concentrations of the supernatants were measured using the Pierce BCA Protein Assay Kit. Supernatant with 1500 μg proteins was then mixed with anti-TRPM4 antibody for 3 h and protein A/G magnetic beads overnight, followed by 3 washes with immunoprecipitation buffer. The precipitates were subsequently boiled in 2x Laemmli buffer and kept at −20°C until use. All other samples were harvested in the immunoprecipitation buffer with protease inhibitor cocktail and 30 μg protein were loaded into each lane for immunoblot analysis.

#### Assessment of disease progression in SOD1^G93A^ mice

Bodyweight and neurological scores were examined weekly starting at the onset of symptoms at week 15, which has been defined by previous studies with the same mouse line.^33^^,^^34^ Neurological scores of the mice were recorded as 0–4 based on the behavior of their hind limbs.^46^ The humane endpoint was defined as the mouse’s inability to rectify itself in 30 s and examined daily from week 19 without knowing the treatment group.

#### Open field test for SOD1^G93A^ mice

Before experiments, mice were habituated to the experimenter and testing room by gentle handling for 1 min in 3 consecutive days. A brief video was taken in the behavior room where the SOD1^G93A^ mice were placed in an open chamber. On the experimental day, the mice were placed into an open arena (50 cm × 50 cm × 50 cm) for a total of 10 min experiment. The total traveled distance of each mouse was traced and analyzed with SYGNIS software, and also the rearing time was reordered without knowledge of the treatment.

#### Immunohistochemistry

The survival of motor neurons was examined by quantifying the motor neuron marker, choline acetyltransferase (ChAT)-positive neurons, and the inflammation was examined by quantifying the microglial cell marker, ionized calcium binding adaptor molecule 1 (Iba1), in the ventral horn of lumbar spinal cord (L3-L5). All control mice and SOD1 mice were perfused with 20 mL PBS followed by 20 mL of 4% paraformaldehyde in PBS. The lumbar spinal cord was dissected and post-fixed in the same solution for an additional 10 min, followed by 24 h incubation with 30% sucrose solution in PBS. Then the spinal cords were snap frozen on dry ice in cryoprotectant solution and 30 μm cryosections were prepared for staining. Floating slices were blocked and permeabilized with the blocking solution (5% donkey serum, 3% bovine serum albumin (BSA), and 0.3% Triton X-100 in PBS) for 1 h and then incubated with either anti-ChAT (1:300) or anti-Iba1 (1:300) antibodies in blocking buffer for 24 h at 4°C. The slices were washed with 1% Triton X-100 in PBS and stained with corresponding secondary antibodies. Images were obtained using EVOS M7000 Imaging System equipped with a 10x objective. For analysis, ChAT-positive cells were manually labeled with ImageJ in 10 ventral horns (5 sections) from the L3-L5 region of each animal. The areas for all ChAT-positive cells were calculated and shown as size distribution, and only areas >300 μm^2^ (diameter >10 μm) were used for motor neuron quantification. Iba1-positive cells were counted manually in at least 6 randomly selected blocks (100 μm^2^) from 3 ventral horns per mice and shown as numbers per 100 μm^2^ per animal. Data analysis was performed without knowledge of treatment.

The analysis of forebrain organoid cell types that undergo NMDA-induced cell death was done using immunostainings of cryosections of 20-week-old forebrain organoids with antibodies against the neuronal markers, NeuN and Myelin transcription factor 1 like (Myt1L) protein, and the astrocyte marker, glial fibrillary acidic protein (GFAP) using the following procedure. All organoids were washed with PBS twice and fixed using 4% paraformaldehyde in PBS for 30 min, followed by 24 h incubation with 30% sucrose solution in PBS. Organoids were snap frozen on dry ice in cryoprotectants and 30 μm cryosections were prepared for staining. Slices were blocked with 3% BSA and 0.3% Triton X-100 in PBS for 1 h and then incubated with either anti-NeuN (1:200), anti-Myt1L (1:1000), or anti-GFAP (1:400) antibodies in the blocking buffer for 12 h at 4°C. Slices were washed with 0.3% Triton X-100 in PBS, stained with the corresponding secondary antibodies, and mounted in Mowiol 4–88 solution containing Hoechst 33258. Images were obtained using a Yokogawa CQ1 confocal microscope with a 10x objective, and z series sum projections were produced from a depth of 20 μm with 2.0 μm intervals. Images were stitched with the CellPathfinder software from Yokogawa. Cells stained with NeuN, Myt1L, and Hoechst were automatically counted with ImageJ with the particle analyzer with a self-developed macro using the Binary-Watershed function, size exclusion was set at 60 μm^2^. NeuN-positive and Myt1L-positive post-mitotic neurons were counted in confocal section z-stacks of whole organoids (5–8 organoids from 2 batches) with ImageJ with the particle analyzer with a self-developed macro using the Binary-Watershed function, size exclusion was set at 60 μm^2^ and expressed as a percentage of total Hoechst-stained nuclei. GFAP staining was quantified by measuring the fluorescent signals of z-stacks of confocal sections of whole organoids (5–6 organoids from 2 batches) using ImageJ and expressed as arbitrary units. Data analysis was performed without knowledge of treatment.

#### Hematology, clinical chemistry and neurofilament light chain

All hematology and most serum clinical chemistry analyses were carried out by IDEXX Laboratories, Germany. The neurofilament light chain levels in serum were determined by PBL Assay Science (USA) using the Simoa NF-light Advantage Kit (Quanterix Cat #103186).

#### Pharmacology safety screening of FP802

Pharmacology safety of FP802 was evaluated in radio ligand binding assays carried out by Eurofins Panlabs Discovery Services Taiwan, Ltd. Significant responses are defined as over 50% inhibition or stimulation in the primary assays. No significant results were noted with FP802.

#### Automated patch clamp

Recombinant human GluN1 (UniProt ID Q05586) with GluN2A (Uniprot Q12879) or GluN2B (Uniprot Q13224) NMDARs were stably expressed under tetracycline control in HEK293 cell lines (Charles River). Voltage-clamp recordings were performed using the Sophion Qube platform which performs 384 parallel and independent patch-clamp recordings with digitally controlled microfluidics (Tecan D300) on a disposable single-hole or multi-hole QChip. The following extracellular solution was used: 145 mM NaCl, 4 mM KCl, 10 mM HEPES, 10 mM glucose, 2 mM CaCl_2_, 1 mM MgCl_2_; pH 7.4. The following intracellular solution was used: 70 mM CsF, 70 mM CsCl, 10 mM HEPES, 1 mM EGTA; 316 mOsm, pH 7.2. For analysis of channel activation kinetics, cells were held at −70 mV and stepped to −20 mV before the application of glycine (100 μM) plus NMDA (90 μM for GluN1/GluN2A and 40 μM for GluN1/GluN2B). These concentrations represent saturating glycine and EC_80_ NMDA concentrations for glutamate receptor activation as determined previously for each cell line. Quality control thresholds were set according to the seal resistance (minimum 20 mΩ), and failed experiments were excluded from the analysis. For analysis of steady state agonist IC_50_ values, voltage steps to +40 mV were performed in the presence and absence of agonists before and after compound application in the same cell. IC_50_ data were generated from agonist responses as described before.^20^

#### NMDA-induced neurotoxicity in human iPSC-derived organoids

The NMDA-induced neurotoxicity assay was carried out with 18–20 week-old organoids and measured in 96-microwell plates with the RealTime-Glo MT Cell Viability Assay (Promega, G9711) on a plate reader set at 5% CO_2_ at 37°C. The MT Cell Viability Substrate can diffuse into cells and only metabolically active cells can reduce the substrate and produce luminescence which is proportional to the live cells in an organoid. Before the toxic stimulation, the organoid was incubated with NanoLuc Enzyme and MT Cell Viability Substrate for 30 min to collect the basal luminescence signal. After NMDA stimulation (0 h), the plate was put in the plate reader and the luminescence signal was collected every 0.5 h for 24 h. The luminescence of each time point F_*(t)*_ was normalized to the basal signal for every single organoid as fold increase, where it reflects the cell viability. The area under the curve (AUC) of F_*(t)*_ over a 24 h period was shown as AUC_0–24h_, and also the viability of organoid cells, which is based on the following equation:

$$Viability\;of\;organoid\;cells\;\left(\%\right)=\frac{{AUC}_{0-24h}}{{AUC}_{control,0-24h}}\times 100\%$$

Where AUC_control, 0–24h_ stands for the data from organoids without any compound or NMDA insult.

### Quantification and statistical analysis

All statistical analyses were performed using GraphPad Prism (RRID: SCR_002798). The statistical details of each experiment can be found in the figure legends, including the statistical tests used, exact value of n, what n represents, definition of center, and dispersion and precision measures. The significance was defined as p < 0.05 in selected statistical methods. For unpaired t test, we used F test to examine the Gaussian distribution; to determine if the p value of the F test is significant, Mann-Whiney test was performed. For one- and two-way ANOVA, we used the Brown-Forsythe test to examine the SD; if the p value of the Brown-Forsythe was significant, Brown-Forsythe and Welch ANOVA tests were used. Exclusion of subjects was clearly indicated in the section of method details. Biostatistical and biometrical planning was guided and reviewed by the Department of Medical Biometry and Informatics at Heidelberg University. *A priori* power calculations of animal numbers were carried out. Sample sizes were calculated using SAS version 9.1 (Statistical Analysis System, RRID: SCR_008567) pro-power to ensure adequate power of key experiments in detecting pre-specified effect sizes.

## Supplemental information

Document S1. Figures S1 and S2 and Tables S1–S3

Document S2. Article plus supplemental information
