# Supplementary material for: TwinF interface inhibitor FP802 stops loss of motor neurons and mitigates disease progression in a mouse model of ALS
Source: Cell Rep Med. 2024 Feb 6;5(2):101413. doi: 10.1016/j.xcrm.2024.101413 (PMC10897598; doi:10.1016/j.xcrm.2024.101413)
Supplement: Document S1. Figures S1 and S2 and Tables S1–S3 [file mmc1.pdf]

Cell Reports Medicine, Volume *5*

Supplemental information

TwinF interface inhibitor FP802 stops loss of motor neurons and mitigates disease progression in a mouse model of ALS

Jing Yan, Yu Meng Wang, Andrea Hellwig, and Hilmar Bading

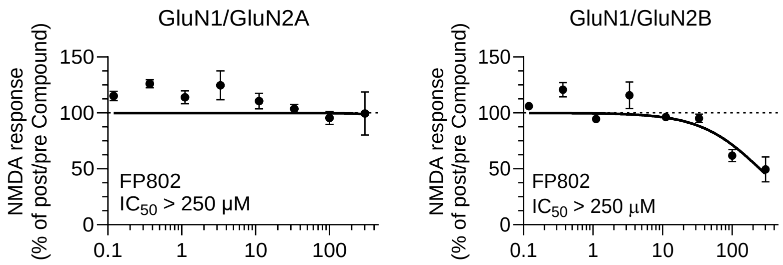

Figure S1. Analysis of IC50 values of FP802 for NMDARs in HEK293 cells. Voltage clamp recordings were performed using the Sophion Qube platform to assess the activation levels of human GluN1/GluN2A or GluN1/GluN2B NMDARs in the presence of FP802. The effects of FP802 on NMDARs were evaluated at +40 mV for eight concentrations (in µM: 0.14, 0.42, 1.2, 3.7, 11.1, 33.3, 100, and 300) in stable HEK293 cell lines expressing human GluN1/GluN2A or GluN1/GluN2B NMDARs. NMDA responses are plotted as the ratio of post/pre compound application and further normalized to 0.3% DMSO as vehicle. IC_{50} estimates were generated from logistic fits of the Hill equation to dose-response relationships. D-APV was used as a control with an IC_{50} value of 4.5 µM and 4.3 µM for GluN1/GluN2A and GluN1/GluN2B, respectively. Data represent mean ± SEM, n = 3-7 from two independent experiments. Related to Figure 1.

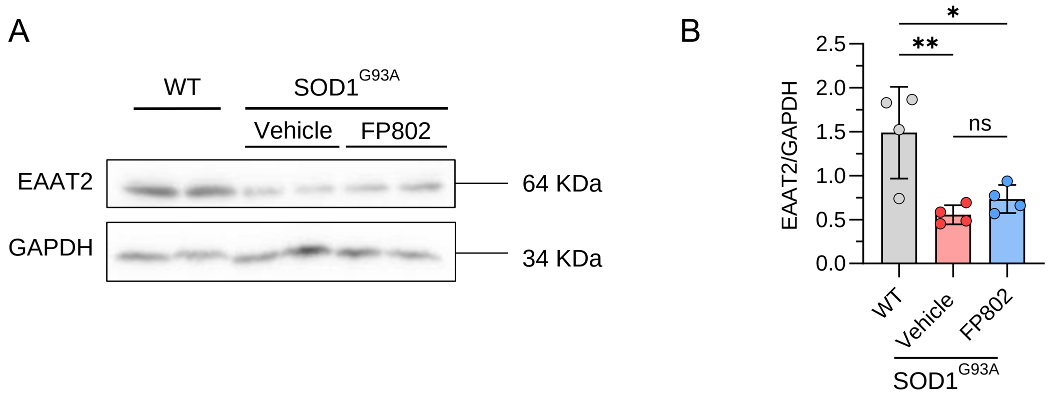

Figure S2. FP802 treatment does not affect EAAT2/GLT1 expression. Immunoblot analysis of EAAT2 and GAPDH expression in the lumbar spinal cord of 19-week-old wild type (WT) and in SOD1^{G93A} mice treated for 4 weeks with vehicle or FP802 (40 mg/kg/day) starting at week 15. (**A**) Representative immunoblots and (**B**) quantitative analysis. Data represent means ± SD, n = 4; ns: no significance, *p < 0.05, **p < 0.01, one-way ANOVA followed by Tukey's multiple-comparisons test. Related to Figure 2.

Assay	ASCII assay name	Ligand or substrate	Species	Tissue/Cell	% inhibition	Time	Temperature
**200510**	Adenosine A1	[3H] DPCPX	Human	recombinant	***-10.6***	90	25
**200610**	Adenosine A2A	[3H] CGS-21680	Human	recombinant	***8.7***	90	25
**203110**	Adrenergic alpha1A	[3H] Prazosin	Human	recombinant	***-12.0***	60	25
**203210**	Adrenergic alpha1B	[3H] Prazosin	Human	recombinant	***-6.3***	60	25
**203630**	Adrenergic alpha2A	[3H] Rauwolscine	Human	recombinant	***1.4***	60	25
**204010**	Adrenergic beta1	[125I] Cyanopindolol	Human	recombinant	***-10.5***	120	25
**204110**	Adrenergic beta2	[3H] CGP-12177	Human	recombinant	***-4.7***	60	25
**204410**	Transporter, Norepinephrine (NET)	[125I] RTI-55	Human	recombinant	***-6.9***	180	4
**214600**	Calcium Channel L-Type, Dihydropyridine	[3H] Nitrendipine	Rat	cerebral cortex	***-3.0***	90	25
**217050**	Cannabinoid CB1	[3H] SR141716A	Human	recombinant	***3.5***	60	37
**219500**	Dopamine D1	[3H] SCH-23390	Human	recombinant	***-1.6***	120	37
**219700**	Dopamine D2S	[3H] Spiperone	Human	recombinant	***-7.2***	120	25
**226500**	GABAA, Muscimol, Central	[3H] Muscimol	Rat	brain (minus cerebellum)	***-7.6***	10	4
**226600**	GABAA, Flunitrazepam, Central	[3H] Flunitrazepam	Rat	brain (minus cerebellum)	***-0.3***	60	25
**233000**	Glutamate, NMDA, Phencyclidine	[3H] TCP	Rat	cerebral cortex	***-5.0***	45	25
**239610**	Histamine H1	[3H] Pyrilamine	Human	recombinant	***20.5***	180	25
**241000**	Imidazoline I2, Central	[3H] Idazoxan	Rat	brain (minus cerebellum)	***-9.2***	30	25
**252710**	Muscarinic M2	[3H] N-Methylscopolamine	Human	recombinant	***1.0***	120	25
**252810**	Muscarinic M3	[3H] N-Methylscopolamine	Human	recombinant	***-5.5***	120	25
**258700**	Nicotinic Acetylcholine Alpha1, Bungarotoxin	[125I] alpha-Bungarotoxin	Human	RD cells	***12.0***	120	25
**258730**	Nicotinic Acetylcholine alpha3beta4	[125I] Epibatidine	Human	recombinant	***-6.3***	60	25
**260410**	Opiate mu (OP3, MOP)	[3H] Diprenorphine	Human	recombinant	***11.0***	60	25
**264500**	Phorbol Ester	[3H] PDBu	Mouse	brain (minus cerebellum)	***21.6***	60	25
**265600**	Potassium Channel [KATP]	[3H] Glyburide	Hamster	pancreatic HIT-T15 beta cells	***-3.9***	120	25
**265900**	Potassium Channel hERG	[3H] Astemizole	Human	recombinant	***-18.4***	60	25
**268420**	Prostanoid EP4	[3H] Prostaglandin E2 (PGE2)	Human	recombinant	***3.7***	120	25
**270000**	Rolipram	[3H] Rolipram	Rat	brain	***-17.9***	60	4
**271700**	Serotonin (5-Hydroxytryptamine) 5-HT2B	[3H] Lysergic acid diethylamide (LSD)	Human	recombinant	***2.3***	60	37
**279510**	Sodium Channel, Site 2	[3H] Batrachotoxinin	Rat	brain (minus cerebellum)	***-14.5***	60	37
**299034**	Sigma1	[3H] Pentazocine	Human	Jurkat cells	***31.3***	120	37

Table S1. Pharmacology safety profile of FP802.
No significant binding activities of FP802 (10 µM) were found in the above-listed assays. Related to Figure 1.

Hematology		SOD1^{G93A}				Reference
		Vehicle	FP802	n	Significance	95% Interval
Erythrocytes (Red blood cells)	Erythrocyte (T/L)	10.70 ± 0.28	10.08 ± 0.38	4	no	7.14-12.20
	Reticulocyte (%)	4.92 ± 0.32	5.10 ± 0.81	4	no	-
	Hematocrit (%)	53.50 ± 3.11	50.25 ± 1.71	4	no	37.3-62.0
	Anisocytosis (%)	0	0	4	n.a.	-
Leukocytes (White blood cells)	Leukocytes (G/L)	2.75 ± 0.51	3.53 ± 1.32	4	no	4.45-13.96
	Lymphocyte (%)	70.75 ± 6.95	71.50 ± 5.57	4	no	-
	Monocyte (%)	4.25 ± 2.22	4.25 ± 1.26	4	no	-
	Segmented neutrophils (%)	24.50 ± 9.43	22.50 ± 6.40	4	no	-
	Band neutrophils	0	0	4	n.a.	-
	Basophils (%)	0	0	4	n.a.	-
	Eosinophils (%)	0.50 ± 1.00	0.25 ± 0.50	4	no	-
Other types of cells	Thrombocytes (G/L)	1490.00 ± 188.10	1506.00 ± 174.68	4	no	841-2159
	Atypical cells	0	0	4	n.a.	-
Other parameters	MCV (fL)	50.00 ± 1.41	49.75 ± 0.50	5	no	42.7-56.0
	MCHC (g/dL)	27.75 ± 0.96	28.50 ± 0.58	5	no	24.6-34.9
	MCH (pg)	13.85 ± 0.19	14.00 ± 0.12	5	no	11.7-16.3
	Hemoglobin (g/dL)	14.83 ± 0.46	14.13 ± 0.68	5	no	10.8-19.2

Table S2. Hematology of SOD1^{G93A} mice treated with vehicle or FP802.
FP802 delivered with a subcutaneously implanted ALZET osmotic pump at 40 mg/kg/day for 4 weeks starting at 15 weeks of age had no adverse effects on the hematology of SOD1^{G93A} mice. References were from Charles River Laboratories datasheets for C57BL6 mice. Related to Figure 2.

Serum clinical chemistry		SOD1^{G93A}				Reference
		Vehicle	FP802	n	Significance	95% Interval
Liver function-related parameters	Albumin (g/L)	26.80 ± 2.17	28.00 ± 1.00	5	no	28-38
	Alkaline phosphatase (U/L)	80.80 ± 22.72	69.40 ± 6.84	5	no	111-275
	ALT (U/L)	35.20 ± 3.42	32.60 ± 8.30	5	no	28-129
	AST (U/L)	177.20 ± 74.85	195.20 ± 147.29	5	no	46-392
	GGT	Not detectable	Not detectable	5	n.a.	0-8
Ions	Calcium (mmol/L)	2.38 ± 0.08	2.35 ± 0.06	5	no	2.4-3.2
	Potassium (mmol/L)	7.50 ± 3.17	9.02 ± 3.83	5	no	7.6-11.2
	Sodium (mmol/L)	151.8 ± 2.5	149.6 ± 3.9	5	no	145.2-176.2
	Chloride (mmol/L)	112.00 ± 1.00	111.20 ± 3.03	5	no	110.7-129.8
	Inorganic phosphate (mmol/L)	3.22 ± 0.61	3.26 ± 0.18	5	no	2.6-4.7
Kidney function-related parameters	Creatine kinase (U/L)	1265 ± 1506	1500 ± 1475	5	no	-
	Creatinine (µmol/L)	8.00 ± 3.56	9.00 ± 2.16	4	no	17.7-44.2
Other parameters	Cholesterol (mmol/L)	2.52 ± 0.86	2.68 ± 0.60	5	no	1.8-4.4
	LDH (U/L)	699.5 ± 337.0	813.3 ± 474.5	5	no	-
	Lipase (U/L)	33.25 ± 7.89	32.80 ± 4.15	4	no	-
	Total protein (g/L)	48.20 ± 2.95	50.00 ± 0.71	5	no	48-70
	Triglycerides (g/L)	0.62 ± 0.33	0.48 ± 0.13	5	no	0.67-2.78
	Urea (BUN) (mmol/L)	7.25 ± 1.02	7.58 ± 1.32	5	no	2.5-10.0

Table S3. Clinical chemistry serum parameters of SOD1^{G93A} mice treated with vehicle or FP802.
FP802 delivered with a subcutaneously implanted ALZET osmotic pump at 40 mg/kg/day for 4 weeks starting at 15 weeks of age had no adverse effects on the serum clinical chemistry parameters of SOD1^{G93A} mice. References were from Charles River Laboratories datasheets for C57BL6 mice. Related to Figure 2.
